# Phenotyping of tako tsubo cardiomyopathy – structural comparison to acute myocardial infarction

**DOI:** 10.1186/1532-429X-11-S1-P2

**Published:** 2009-01-28

**Authors:** Andreas Rolf, Guido Conradi, Johannes Rixe, Holger Steiger, Holger Nef, Helge Möllmann, Katharina Beiring, Christian Hamm, Thorsten Dill

**Affiliations:** Kerckhoff-Heart-Center, Bad Nauheim, Germany

**Keywords:** Acute Myocardial Infarction, Acute Myocardial Infarction, Acute Myocardial Infarction Patient, Tako Tsubo Cardiomyopathy, Short Axis Slice

## Objective

The tako tsubo cardiomyopathy (TTC) is characterized by a transient contractile dysfunction after severe physical or emotional stress. Relevant coronary artery disease is absent. Clinically it mimics acute myocardial infarction (AMI). Therefore, the aim of this study was to characterize morphological differences between TTC and AMI using different MRI parameters.

## Methods

22 TTC and 35 AMI patients were examined within 48 hours upon admission. Ejection fraction (EF), myocardial mass (MM) enddiastolic (EDV) and endsystolic volumes (ESV) and regional contractility scores were computed on 10 contiguous CINE SSFP short axis slices. Myocardial edema (ME) was computed on T2 weighted TSE images on short axis orientations, late enhancement (LE) was evaluated on FLASH 3D GRE (both defined as signal intensity of more than 2 standard deviations from remote myocardium). Measurements are given as mean ± SE. Differences were computed using an ANOVA model, a p value =< 0.05 was cosidered significant.

## Results

All AMI patients and none of the TTC patients included had LE. TTC patients had a significantly lower EF (TTC 43.8 ± 2.4%; AMI 50.6 ± 1.9%; p = 0.03) all other comparisons were therfore controlled for EF. TTC patients had significantly smaller ventricular volumes (EDV – TTC: 138 ± 5.6 ml; AMI: 161 ± 6.0 ml; p = 0.24; ESV-TTC: 72.9 ± 4.5 ml; AMI: 86.6 ± 3.8; p = 0.9) myocardial mass (TTC: 102.8 ± 7.1 ml; AMI: 140 ± 5.6; p = 0.0001) and edema (TTC: 19.5 ± 4.2%; AMI: 27.2 ± 2.6%; p = 0.13). In contrast regional contractility was more affected in TTC with a significantly larger number of dysfunctional segments (TTC: 7.5 ± 0.6 segments; AMI: 4.3 ± 0.5 segments; p = 0.0001) and a significantly higher wall motion score (TTC: 1.9 ± 0.1; AMI: 1.4 ± 0.1; p = 0.0001).

## Conclusion

In contrast to AMI, TTC patients show a marked regional dysfunction but no signs of early remodeling like ventricular enlargement and increased myocardial mass.

